# Sharp-Wave EEG Activity and Cytomegalovirus Exposure in Schizophrenia Spectrum Disorders: A Neuroimmune Perspective

**DOI:** 10.3390/jcm15124841

**Published:** 2026-06-22

**Authors:** Mădălina Georgeta Sighencea, Marius Cornițescu, Simona Corina Trifu

**Affiliations:** 1 Doctoral School, “Carol Davila” University of Medicine and Pharmacy, 020021 Bucharest, Romania; madalina-georgeta.iliescu@drd.umfcd.ro; 2 Department of Functional Sciences, “Carol Davila” University of Medicine and Pharmacy, 020021 Bucharest, Romania; marius.cornitescu@umfcd.ro; 3 Department of Clinical Neurosciences, “Carol Davila” University of Medicine and Pharmacy, 020021 Bucharest, Romania

**Keywords:** schizophrenia spectrum disorders, cytomegalovirus, electroencephalography (EEG), sharp-wave activity, resting-state EEG, neuroimmune mechanisms, immune dysregulation, cognitive impairment, treatment-resistant schizophrenia

## Abstract

**Background**: Immune mechanisms are increasingly implicated in the heterogeneity of schizophrenia spectrum disorders. Cytomegalovirus (CMV), a latent immunomodulatory herpesvirus, is linked to cognitive and immunological alterations, but its electrophysiological correlates remain largely unexplored. This study investigates the relationships among CMV serostatus, EEG features, inflammatory markers, and clinical–cognitive variables. **Methods**: In this prospective cross-sectional study, 123 patients with schizophrenia spectrum disorders underwent integrated clinical, cognitive, laboratory, and qualitative visual EEG assessments. CMV exposure was determined via IgG serology. **Results**: Global electroencephalographic EEG organization did not differ by CMV serostatus. However, a descriptive increase in resting-state sharp-wave discharges was observed in CMV-seronegative patients, independent of baseline cortical rhythms. Immunologically, CMV-seropositive individuals exhibited significantly higher total leukocyte counts, consistent with latent viral immune remodeling rather than overt systemic inflammation. Clinically, CMV-seropositive patients demonstrated descriptively higher scores on the disorganization dimension derived from the PANSS (Positive and Negative Syndrome Scale) five-factor consensus model. While these variations did not retain statistical significance after multiple testing correction, separate dimensional analyses revealed that patients exhibiting sharp waves demonstrated better overall cognitive functioning and superior performance within a memory-related item grouping. Notably, the presence of sharp-wave activity was independent of both peripheral inflammatory profiles and treatment-resistant status, underscoring a distinct electrophysiological phenotype. **Conclusions**: CMV exposure represents a modulating biological background associated with corrected leukocyte elevations and subtle electrophysiological variability, rather than a direct determinant of global clinical severity. The nominal EEG variations and their independent link to better-preserved memory performance highlight non-linear neuroimmune interactions. Given the cross-sectional design, these exploratory patterns warrant a non-causal interpretation but outline a foundation for future longitudinal investigations.

## 1. Introduction

Understanding psychotic disorders remains a formidable challenge in psychiatry, largely because traditional diagnostic frameworks fail to capture their pronounced biological and clinical heterogeneity [1,2,3]. Schizophrenia spectrum disorders, as delineated in the Diagnostic and Statistical Manual of Mental Disorders, Fifth Edition, Text Revision (DSM-5-TR), comprise conditions defined by pervasive distortions in perception, thought, and behavior that severely compromise adaptive functioning [1,4]. While schizophrenia itself affects less than 1% of the global population and stands as a major driver of long-term disability [1,2], schizophreniform disorder presents with equivalent neurobiological and clinical features but is distinguished strictly by its shorter duration of one to six months [4]. Decades of intensive research have only partially clarified the mechanisms driving this phenotypic variability, underscoring the need for integrative pathophysiological models [1,2,3].

Instead of focusing on localized structural abnormalities, contemporary neurobiology increasingly frames these disorders as systemic disturbances in large-scale brain organization, rooted in aberrant neurodevelopment and faulty synaptic plasticity [1,2,5]. Emerging neuroimaging and electrophysiological data point to a breakdown in coordination across distributed neural networks, reflecting an excitation–inhibition imbalance within core cortico-thalamo-cortical circuits [6,7]. At the systems level, neural oscillations act as the primary vehicle for inter-regional communication; alpha and beta rhythms, in particular, preserve network stability by tuning these inhibitory and excitatory interactions [7,8,9]. Desynchronization in these oscillatory patterns reflects altered circuit regulation and functional dysconnectivity rather than static structural lesions [10,11,12]. Within this framework, transient paroxysmal graphoelements, such as sharp-wave activity, can be interpreted as dynamic fluctuations in network excitability driven by circuit-level anomalies, rather than signs of classic epileptiform pathology [5,12,13,14]. Yet, why these electrophysiological patterns vary so drastically between individual patients remains an open question [1,3,5].

Given that oscillatory synchronization depends on precise synaptic homeostasis, biological systems capable of modifying synaptic function—most notably immune signaling—have become prime candidates for explaining this electrophysiological diversity [5,15]. Schizophrenia is now widely viewed through a neurodevelopmental, multi-hit lens, where genetic susceptibility interacts with environmental immune disruptions [1,16]. Peripheral inflammatory signals can cross-talk with the central nervous system via cytokine-mediated pathways, actively shaping microglial synaptic remodeling, neurotransmission, and network dynamics [15,17,18]. Because these inflammatory cascades directly modulate the dopaminergic and glutamatergic pathways central to the illness [3,19], they support the hypothesis that a low-grade neuroinflammatory state defines a biologically distinct patient subgroup [20,21].

Consequently, neurotropic viruses that induce chronic immune dysregulation—particularly within the herpesvirus family—hold significant interest due to their capacity for lifelong latency and persistent immunomodulation [22]. Cytomegalovirus (CMV) establishes persistent infections that drive systemic immune remodeling, characterized by the expansion of differentiated memory T-cells and altered cytokine networks, even in entirely asymptomatic hosts [23,24,25,26,27]. A growing body of work suggests that CMV impacts central function through indirect neuroimmune and neurodevelopmental trajectories rather than direct cytopathology. Serological data link CMV exposure to severe negative symptoms and neurocognitive deficits, while experimental models indicate that viral gene expression during development can disrupt neuronal migration and synaptic architecture [28,29,30]. These insights are reinforced by epidemiological associations linking early infectious exposures and autoimmune disorders to a heightened risk of developing schizophrenia-spectrum conditions [31].

Despite these clues, empirical research has historically remained fragmented, focusing almost exclusively on either the immune, clinical, or cognitive outcomes of CMV exposure [24,29]. As a result, the relationship between viral serostatus and cortical electrophysiological organization remains fundamentally unexplored.

While acute, fulminant central nervous system CMV infections characteristically present with diffuse EEG slowing or frank epileptiform discharges—clear markers of global inflammatory tissue damage [32,33,34]—the subtle impact of latent, low-grade infection requires a distinct conceptual framework. Mechanistically, latent CMV may modulate cortical excitability not through direct viral cytopathology, but via sustained, low-grade peripheral immune remodeling and systemic cytokine shifts [25,26,27,28]. This systemic inflammatory state can chronically alter blood–brain barrier permeability and trigger low-grade microglial activation within the central nervous system [15,17]. These activated cells, in turn, disrupt synaptic homeostasis and interfere with local neurotransmission—particularly by modulating GABAergic and glutamatergic pathways central to the illness [3,18,19]. This continuous neuroimmune cross-talk chronically shifts the cortical excitation–inhibition balance, altering the threshold for transient paroxysmal activity, such as sharp waves, within the vulnerable networks of psychiatric populations [5,12,32].

Progress in contemporary psychiatry relies on breaking down these silos and integrating electrophysiological, immunological, and clinical–cognitive metrics within unified analytical frameworks capable of capturing real-world biological heterogeneity [1,2]. Resolving this blind spot is essential to determine whether latent viral exposure serves as a clinically meaningful modifier of cortical network regulation [1,2]. To address this, the present study evaluates the relationships among cytomegalovirus serostatus, EEG parameters, peripheral inflammatory markers, and clinical–cognitive characteristics in individuals with schizophrenia spectrum disorders. By mapping these electrophysiological, immunological, and dimensional clinical variables within a single cohort, we aim to clarify whether prior CMV exposure patterns are tied to specific variations in brain network functioning, immune signaling, and clinical–cognitive phenotypes.

## 2. Materials and Methods

### 2.1. Study Design and Participants

This hospital-based prospective cross-sectional study was conducted at the “Prof. Dr. Alexandru Obregia” Clinical Hospital of Psychiatry, Bucharest, Romania, between 2024 and 2025. All eligible patients admitted during the study period were consecutively invited to participate. Only individuals who provided written informed consent, or for whom written informed consent was obtained from a legal guardian (or legally authorized representative, in cases of involuntary admission or compromised decision-making capacity) were included. The prospective cross-sectional design involved standardized clinical, neurophysiological, and laboratory assessments performed during the same hospitalization period, allowing integrated evaluation of clinical and biological variables at a single observational time point.

**Figure 1:**
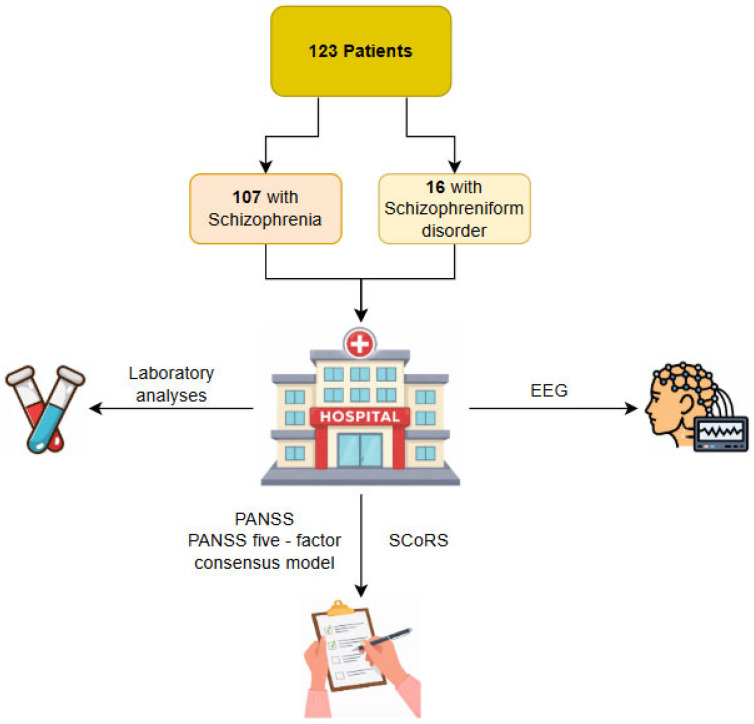
Study workflow. EEG—electroencephalography; PANSS—Positive and Negative Syndrome Scale; SCoRS—Schizophrenia Cognition Rating Scale.

Participants were adults diagnosed with schizophrenia spectrum disorders according to DSM-5-TR criteria [4]. A total of 123 patients were enrolled, including 107 with schizophrenia and 16 with schizophreniform disorder. Schizophreniform disorder was defined as schizophrenia-like psychotic symptoms with a duration between 1 and 6 months, irrespective of episode status. Given their shared psychopathological and presumed neurobiological mechanisms, schizophrenia and schizophreniform disorder were analyzed together as part of the schizophrenia spectrum. Inclusion criteria included: DSM-5-TR diagnosis of schizophrenia or schizophreniform disorder, age between 18 and 70 years, and provision of written informed consent. The selected age range was intended to ensure a relatively homogeneous adult cohort while minimizing potential age-related confounding effects in neurophysiological analyses. Exclusion criteria included: the absence of a schizophrenia spectrum diagnosis, refusal to provide informed consent, age less than 18 years or older than 70 years, and severe medical or neurological conditions that could interfere with the interpretation of EEG or study procedures (e.g., alcohol-related dementia, Alzheimer’s disease). Participants were recruited from inpatient and outpatient psychiatric services—encompassing a wide severity spectrum that included both voluntary and involuntary admissions—as part of an observational research program that investigated clinical, immunological, and electrophysiological correlations of psychotic disorders. The present analyses address predefined research questions within this broader research framework. Figure 1 shows the workflow of the current study.

### 2.2. Clinical and Psychopathological Assessment

All participants underwent a comprehensive clinical evaluation conducted by trained psychiatrists at the time of study inclusion. Sociodemographic and illness-related variables were systematically recorded according to those reported in Section 3 and included age, sex, educational level (low, medium, high), place of residence (urban/rural), smoking status (current smoker/non-smoker), age at illness onset, duration of the illness, number of psychiatric hospitalizations in the previous 12 months, lifetime number of suicide attempts, and diagnosis of schizophrenia or schizophreniform disorder according to the criteria of DSM-5-TR.

Treatment-resistant illness was pragmatically defined as the clinical requirement for clozapine treatment and/or electroconvulsive therapy (ECT), reflecting established indicators of insufficient response to prior antipsychotic therapy in routine practice and consistent with consensus definitions of treatment-resistant schizophrenia proposed by the Treatment Response and Resistance in Psychosis (TRRIP) Working Group [35]. Treatment decisions, including the initiation of clozapine or the administration of electroconvulsive therapy (ECT), were made by treating clinicians according to routine clinical judgment and were not influenced by participation in the study. Information regarding ongoing psychopharmacological treatment was documented at the time of EEG recording. Given the high heterogeneity of treatment regimens and the frequent use of antipsychotic polytherapy, individual medication dosages and specific drug combinations were considered a naturalistic clinical condition rather than distinct analytical factors. However, to account for major clinical phenotypes linked to treatment response, treatment-resistant status—defined as the clinical requirement for clozapine therapy or electroconvulsive therapy (ECT)—was explicitly integrated and adjusted for within the statistical models.

Clinical assessments were conducted under usual treatment conditions, and all evaluations were completed prior to EEG recording to ensure temporal consistency between psychopathological characterization and neurophysiological assessment.

Psychopathological severity was assessed using the Positive and Negative Syndrome Scale (PANSS) [36], administered by the Structured Clinical Interview for PANSS (SCI-PANSS) [37], a 30-item clinician-rated instrument evaluating positive symptoms, negative symptoms, and general psychopathology on a seven-point scale (1 = absent to 7 = extreme), with higher scores indicating greater severity of symptoms; total PANSS scores were calculated for all participants.

Symptom dimensions were further characterized using the PANSS five-factor consensus model [38], which was derived from previously published five-factor solutions of the PANSS, including the model described by Marder et al. [39]. In the present study, factor scores were operationalized as the sum of the predefined PANSS items within each dimension. Based on the distribution of the consensus items [39], the following factors were defined: positive factor (P1, P3, P5, G9), negative factor (N1, N2, N3, N4, N6, G7), disorganized/concrete factor (P2, N5, G11), excited factor (P4, P7, G8, G14), and depressed factor (G2, G3, G6).). This dimensional approach allowed for the characterization of schizophrenia symptom domains beyond the traditional positive–negative dichotomy.

Cognitive functioning was assessed using the Schizophrenia Cognition Rating Scale (SCoRS) [40], an interviewer-based instrument designed to evaluate the impact of cognitive impairments on everyday functioning in schizophrenia spectrum disorders and described in the literature as encompassing multiple cognitive domains, including memory, learning, attention, working memory, problem solving, processing/motor speed, communication/social cognition, and language [41]. Both the SCoRS Global Rating and the SCoRS Total Score were recorded, with higher scores indicating greater cognitive impairment. In addition to standard SCoRS scoring, exploratory item-level analyses were conducted. Individual SCoRS items were grouped into five broader domain-level variables (memory, attention, executive functioning, language, and visuospatial ability) based on their conceptual content and theoretical correspondence with related cognitive constructs described in the literature [41]. This aggregation reflects a consolidation of related domains (e.g., learning and working memory within memory-related processes; problem solving and processing speed within executive functioning) and was performed to facilitate exploratory characterization of domain-related cognitive functioning. These groupings were not intended to represent validated psychometric subscales or to redefine the original domain structure of the instrument, but rather to support exploratory dimensional analyses within the present study.

Following clinical and cognitive evaluation, the participants followed the standardized neurophysiological and laboratory assessment protocol described in subsequent sections, allowing integrated examination of psychopathological severity, cognitive functioning, inflammatory markers, EEG characteristics, serological status of CMV, and treatment-resistant illness within a unified analytical framework. Clinical assessments were completed prior to laboratory sampling and EEG recording to ensure temporal separation between psychopathological evaluation and subsequent biological and neurophysiological measurements.

### 2.3. Electroencephalographic Assessment

Electroencephalographic recordings were prospectively acquired under standardized clinical conditions in a quiet, dimly lit room, with participants in a wakeful resting state. A participant declined EEG examination; therefore, analyses were conducted for all participants with available recordings (*N* = 122). A standard 21-electrode montage was applied according to the international 10–20 system, and the recordings included alternate closed and open eyes to assess background activity of the resting-state and physiological reactivity. Each recording lasted 20 to 30 min. The recordings were continuously monitored to ensure adequate wakefulness and minimize common EEG artifacts, including movement, muscle activity, and eye movements, according to routine clinical neurophysiology practice. When clinically indicated, hyperventilation and intermittent photic stimulation were performed. EEG acquisition followed routine clinical neurophysiology standards, with high-pass and low-pass filters maintained within conventional clinical ranges appropriate for assessment of background rhythms and slow-wave activity, and a 50 Hz notch filter applied to reduce line interference. Minor parameter adjustments were made only to optimize signal quality while remaining within accepted clinical standards. All recordings were analyzed using qualitative visual EEG assessment by an experienced neurologist blinded to clinical and biological data, including CMV serostatus, and interpretation followed predefined categorical criteria consistent with ACNS [42] and IFCN recommendations [43].

Visual clinical EEG assessment was selected because the study aimed to characterize clinically interpretable electrophysiological patterns rather than perform quantitative spectral analysis. Clinical and laboratory assessments were conducted independently of the interpretation of the EEG to minimize potential assessment bias. The analysis systematically evaluated background rhythm characteristics (dominant EEG rhythm, alpha peak frequency, spatial distribution, bilateral symmetry, spindle-like activity of the dominant rhythm, and alpha activity with sharply contoured morphology), beta activity characteristics (presence, amplitude categorized as low, moderate, or high, localization, distribution pattern, and spindle-like beta activity), irritative and epileptiform abnormalities (spontaneous sharp-wave activity during resting-state EEG, sharp waves during hyperventilation, sharp-wave activity overall, spontaneous spike discharges, spontaneous spike–wave complexes, spike or spike–wave activity overall, spike or spike–wave activity during hyperventilation, and spike or spike–wave activity during photic stimulation), as well as slow-wave abnormalities (spontaneous rhythmic delta activity, rhythmic delta activity overall, hyperventilation-induced delta activity, spontaneous rhythmic theta activity, rhythmic theta activity overall, hyperventilation-induced rhythmic theta activity, and delta activity during eyes-closed recording and photic stimulation). Irritative EEG abnormalities and EEG features suggestive of a structural lesion were documented when present. EEG assessment was predefined as a qualitative neurophysiological outcome measure intended to evaluate functional brain alterations potentially associated with CMV exposure and treatment-resistant illness.

### 2.4. Laboratory Assessments

As part of the standardized study protocol, peripheral venous blood samples were obtained from all participants under routine clinical conditions during hospitalization at the “Prof. Dr. Alexandru Obregia” Clinical Hospital of Psychiatry. Complete blood count parameters and inflammatory markers were analyzed in the hospital’s certified clinical laboratory using automated hematology analyzers integrating impedance, flow cytometry, and spectrophotometric measurement principles according to institutional procedures. The analyzed hematological variables included total leukocyte count, absolute and relative neutrophil, lymphocyte, and monocyte counts, as well as erythrocyte sedimentation rate (ESR), measured using the Westergren method as a nonspecific marker of systemic inflammation. The hematological parameters were interpreted within the routine clinical laboratory standards. To further characterize the immune–inflammatory status, derived inflammatory indices were calculated from absolute cell counts obtained from the complete blood count, including the neutrophil-to-lymphocyte ratio (NLR), lymphocyte-to-monocyte ratio (LMR) and platelet-to-lymphocyte ratio (PLR). These composite indices were included as exploratory peripheral markers of systemic inflammatory and immune activation processes. Cytomegalovirus IgG serological status was determined from peripheral venous blood samples analyzed in a certified clinical laboratory in Romania using a chemiluminescent immunoassay (CLIA) performed according to manufacturer-standardized protocols. Antibody titters were interpreted according to laboratory reference ranges, with values < 1 considered negative and values ≥1 considered positive. CMV IgG seropositivity was considered a marker of prior exposure consistent with latent CMV infection rather than acute infection.

All laboratory measurements were obtained prior to EEG recording to ensure temporal comparability between biological and neurophysiological assessments. Inflammatory indices, including NLR, PLR, and LMR, together with CMV IgG serostatus, were analyzed alongside clinical and EEG variables within an integrated exploratory framework. Of the 123 enrolled participants, complete datasets were not available for all assessments due to individual preferences regarding specific study procedures. Electroencephalographic recordings were available for 122 participants, as one patient declined the EEG examination. CMV serological tests were not available for five participants who declined CMV testing. Complete blood count data were missing for one participant, and erythrocyte sedimentation rate values were unavailable for three participants. All analyses were performed using available-case data without imputation of missing values; however, all individuals remained included in the study and contributed data to the analyses when available.

### 2.5. Statistical Analysis

Statistical analyses were conducted according to a predefined analytical framework aligned with study objectives and applied to all clinical, neurophysiological, and laboratory data collected. Data processing was performed using Microsoft Excel with the XLSTAT add-in (Addinsoft SARL, Paris, France) and IBM SPSS Statistics version 20.0 (IBM Corporation, Armonk, NY, USA). Distributional assumptions were assessed using the Anderson–Darling test. As most continuous variables did not meet the normality criteria, nonparametric statistical methods were applied. Continuous variables are presented as medians and interquartile ranges (IQR), while categorical variables are presented as frequencies and percentages (n%). Between-group comparisons for continuous variables were performed using the two-tailed Mann–Whitney U test. In order to assess the influence of confounding factors, such as sex, smoking, treatment resistance, and age, general linear models were performed, and *p*-values were presented alongside the original Mann–Whitney results.

Associations between categorical variables were evaluated using the chi-square (χ^2^) test of independence (χ^2^). Yates’ continuity correction was applied when appropriate in the cases of small, expected cell counts. All statistical tests were two-tailed and statistical significance was defined as *p* < 0.05. Because of the multiple comparisons conducted in our study, we computed the Benjamini–Hochberg adjusted *p*-values for both qualitative and quantitative tests that we performed.

Cramér’s V coefficient was used to assess the strength of association between two nominal variables and was applied to contingency tables, including those with more than two rows and/or columns. Values > 0.50 were considered indicative of a strong association, values between 0.30 and 0.50 of a moderate association, values between 0.10 and 0.30 of a weak association, and values < 0.10 of a negligible association.

Cognitive domain scores derived from SCoRS items were constructed based on a predefined conceptual grouping of items reflecting memory, attention, executive functioning, language and visuospatial domains. Domain scores were calculated as the sum of the corresponding item ratings and were used for exploratory domain-level characterization of cognitive functioning. Because these groupings do not represent validated SCoRS subscales, analyses involving domain scores were considered exploratory and interpreted alongside total and global primary SCoRS scores.

The analyses were structured hierarchically according to predefined study objectives. The primary analysis examined associations between the serostatus of CMV IgG and the characteristics of EEG. Secondary analyses evaluated the relationships between CMV serostatus and inflammatory and clinical variables. Additional analyses exploring associations between EEG features, inflammatory markers, and treatment-resistant illness were considered exploratory and hypothesis-generating. All analyses were performed using available-case data without imputation of missing values; therefore, sample size varied slightly between analyses depending on data availability.

## 3. Results

### Study Population Description

The baseline sociodemographic, clinical, cognitive, and biological characteristics of the study sample are presented in Table 1. A total of 123 patients with psychotic disorders were included in the study. EEG recordings were obtained for 122 participants, as one patient declined the examination. CMV serological testing was available in 118 patients, with five individuals declining this assessment. Complete blood count parameters and derived inflammatory indices were available for 122 participants, while erythrocyte sedimentation rate measurements were missing in three cases due to incomplete laboratory assessment.

The sample was predominantly male (98, 79.67%) and had a median age of 42 years (IQR 33–50). Most participants resided in urban areas (99, 80.48%). Regarding educational level, 81 patients (65.85%) reported a medium level of education, 22 (17.88%) had higher education, and 20 (16.26%) had a low educational level. Current smoking was reported by 79 patients (64.22%).

The median age at illness onset was 25 years (IQR 20–34), and the median duration of the illness was 11 years (IQR 4–21). During the previous 12 months, patients had a median of two psychiatric hospitalizations (IQR 1–4), while the lifetime number of suicide attempts had a median value of 0 (IQR 0–1). According to DSM-5-TR diagnostic criteria, schizophrenia was present in 107 cases (86.99%), while 16 patients (13.01%) met the criteria for schizophreniform disorder. Treatment-resistant illness was identified in 39 patients (31.71%).

Psychopathological severity indicated a substantial symptom burden, with a median PANSS total score of 135 (IQR 114–149). The median scores in the PANSS five-factor consensus model were 22 (IQR 17–25) for the positive factor, 24 (IQR 18–29) for the negative factor, 14 (IQR 10–17) for the disorganized/concrete factor, 19 (IQR 14–24) for the excited factor, and 12 (IQR 10–14) for the depressed factor.

Cognitive functioning assessed using the Schizophrenia Cognition Rating Scale (SCoRS) showed a median global rating of 8.5 (IQR 7.5–9) and a total score of 69 (IQR 61–73). Exploratory domain-level scores suggested moderate impairment in memory (3.2 [2.6–3.4]), attention (4 [3.75–4]), executive functioning (3.8 [3.4–4]), language (3.25 [3–3.5]), and visuospatial abilities (2.5 [2,3]).

Inflammatory and immune markers were within expected clinical ranges overall. The median leukocyte count was 6.96 × 10^3^/µL (IQR 5.86–8.63). The median neutrophil counts were 3.79 × 10^3^/µL (IQR 3.08–4.96), which corresponds to 55.9% (IQR 49.82–62.7). Lymphocyte counts were 2.21 × 10^3^/µL (IQR 1.78–2.77), corresponding to 32.45% (IQR 25.68–38.13), while monocyte counts were 0.59 × 10^3^/µL (IQR 0.47–0.73), corresponding to 8.3% (IQR 6.88–9.8). Derived inflammatory indices included a median neutrophil-to-lymphocyte ratio (NLR) of 1.72 (IQR 1.29–2.50), a lymphocyte-to-monocyte ratio (LMR) of 3.84 (IQR 3.10–4.54), and a platelet-to-lymphocyte ratio (PLR) of 114.67 (IQR 80.78–146.08). The median erythrocyte sedimentation rate was 10 mm/h (IQR 4–14). CMV IgG seropositivity was observed in 103 patients (87.29%), while 15 (12.71%) were CMV-seronegative.

## 4. EEG Characteristics

The distribution of EEG characteristics is presented in Table 2. Background activity was largely preserved, with the alpha rhythm as the dominant pattern in 96.72% of recordings and a mean alpha peak frequency of 9.83 ± 1.1 Hz. The dominant rhythm was most frequently distributed over centrotemporal (47.11%) and parietotemporal regions (52.07%), with bilateral symmetry observed in 88.43% of cases. Spindle-like modulation of the dominant rhythm was present in 40.16% of recordings, while sharply contoured alpha morphology was uncommon (4.92%). Beta activity was nearly ubiquitous (98.36%), frequently of high amplitude (51.67%), and predominantly frontocentrally localized (85.47%), with a primarily temporal distribution. Spindle-like beta modulation was rare (0.82%). Sharp-wave activity was identified in 27.04% of patients overall, including spontaneous resting-state sharp waves in 21.31% and hyperventilation-associated sharp waves in 9.01%, was predominantly centrotemporal (63.64%), and a descriptive review of recordings indicated that these discharges were most commonly bilaterally synchronous. Less frequent topographies included parietotemporal (18.18%) and centroparietotemporal (9.09%) distributions, with isolated frontocentrotemporal, central, and left occipitotemporal patterns (each 3.03%).

Epileptiform abnormalities were rare (0.82%), and overall spike or spike–wave activity was observed in 7.38% of cases. Slow-wave activity was less prevalent. Rhythmic delta activity was present in 13.93% of patients overall, with spontaneous rhythmic delta observed in 10.66% and hyperventilation-induced delta in 4.92%. Delta activity most frequently involved centrotemporal and frontocentral regions and was commonly bilateral. Rhythmic theta activity was observed in 19.67% of patients (16.39% spontaneous), most commonly localized to centrotemporal (40.91%) and central regions (18.18%); these patterns were predominantly bilateral and synchronous on visual inspection. Delta activity during eyes-closed recording and photic stimulation was rare (0.82%). These descriptive EEG findings provided the basis for subsequent analyses examining associations between electrophysiological patterns and clinical and biological variables.

### 4.1. EEG Characteristics According to CMV IgG Serostatus

The associations between cytomegalovirus IgG serostatus and electroencephalographic characteristics were examined using chi-square tests of independence, with Cramér’s V coefficients reported as measures of effect size (see Table 3). Yates continuity correction was applied for small, expected cell counts, and *p*-values were subsequently adjusted using the Benjamini–Hochberg procedure (*p*_BH_) to account for multiple comparisons.

The parameters reflecting background rhythm organization did not differ significantly between CMV-seropositive and CMV-seronegative patients. The distribution of the dominant EEG rhythm was not statistically significant (*p*_BH_ = 0.775). Similarly, alpha peak frequency categories did not show a significant association with the serostatus of CMV (*p*_BH_ = 0.464), and the spatial distribution of the dominant rhythm was comparable between the groups (*p*_BH_ = 0.578). The bilateral symmetry of the dominant (*p*_BH_ = 0.959) and the spindle-like activity of the dominant rhythm (*p*_BH_ = 0.667) were also similar between the groups. Within background rhythm morphology, alpha activity with sharply contoured morphology demonstrated a strong trend toward an association with CMV serological status, being observed more frequently among CMV-seronegative patients (20.00%) compared with CMV-seropositive patients (2.94%). While this association achieved nominal significance using Yates correction (*p* = 0.030), it did not survive Benjamini–Hochberg adjustment (*p*_BH_ = 0.435).

Beta activity characteristics showed no statistically significant associations with CMV serological status, including presence of beta activity (*p*_BH_ = 0.468), beta amplitude distribution (*p*_BH_ = 0.661), beta localization (*p*_BH_ = 0.682), beta distribution pattern (*p*_BH_ = 0.595), and spindle-like beta activity (*p*_BH_ = 0.883).

Analysis of irritative and epileptiform EEG abnormalities revealed a strong trend toward an association between CMV serostatus and spontaneous sharp-wave activity (resting-state EEG), which was notably more frequent among CMV-seronegative patients (53.33%) than among CMV-seropositive patients (16.67%). While this difference achieved high nominal significance prior to multiple comparisons correction (Yates-corrected *p* = 0.003), it retained only a marginal significance after Benjamini–Hochberg adjustment (*p*_BH_ = 0.087).

When sharp-wave activity was analyzed overall, including both spontaneous and activation-related occurrences, a notable difference was observed between groups, with occurrences being more frequent among CMV-seronegative patients (53.33% vs. 23.53%). Although this overall association achieved nominal significance prior to multiple comparisons correction (Yates-corrected *p* = 0.035), it did not survive Benjamini–Hochberg adjustment (*p*_BH_ = 0.338).

Irritative EEG abnormalities and spike or spike–wave activity during hyperventilation demonstrated nominal trends prior to multiple comparisons correction (Yates-corrected *p* = 0.089 and Yates-corrected *p* = 0.051, respectively). However, neither trend remained apparent following Benjamini–Hochberg adjustment (*p*_BH_ = 0.516 and *p*_BH_ = 0.370, respectively).

The analysis of EEG features suggestive of a structural lesion yielded no statistically significant results (*p*_BH_ = 0.512). Delta activity during eyes-closed recording and photic stimulation was also not statistically significant (*p*_BH_ = 0.638).

All remaining EEG parameters, including epileptiform EEG abnormalities, spontaneous spike discharges, spontaneous spike–wave complexes, spike or spike–wave activity during photic stimulation, and rhythmic delta or theta activity that occurs spontaneously or during activation procedures, did not show a statistically significant association with CMV serological status (all *p*_BH_ > 0.05).

In general, notable trends were limited to a subset of paroxysmal EEG abnormalities—specifically alpha activity with sharply contoured morphology and spontaneous sharp-wave activity—both demonstrated a higher nominal prevalence among CMV-seronegative patients. Conversely, background rhythm organization, beta activity characteristics, and slow-wave activity showed no association with CMV serostatus, even prior to multiple comparisons correction.

### 4.2. Inflammatory and Immune Markers According to CMV Serostatus

Inflammatory and immune markers stratified by CMV serostatus are summarized in Table 4. At the univariable screening level, CMV-seropositive patients demonstrated significantly higher total leukocyte counts compared with CMV-seronegative individuals (median 7.24 vs. 5.77 × 10^3^/µL; unadjusted *p* < 0.001). Crucially, this robust finding successfully maintained its statistical significance both after adjustment for confounders (age, sex, smoking status, and treatment resistance) and following strict multiple comparisons correction (confounder-adjusted *p* [*p*_adj_] = 0.002, Benjamini–Hochberg adjusted *p* [*p*_BH_]= 0.045).

This global leukocyte elevation was independently mirrored by increased absolute neutrophil counts (median 4.01 vs. 3.35 × 10^3^/µL; unadjusted *p* = 0.014, *p*_adj_ = 0.023) and absolute lymphocyte counts (median 2.30 vs. 1.75 × 10^3^/µL; unadjusted *p* = 0.002, *p*_adj_ = 0.008). While these specific leukocyte subsets demonstrated strong nominal associations with CMV seropositivity that resisted confounding control, they did not survive the conservative false discovery rate restriction, shifting to non-significant trends following Benjamini–Hochberg adjustment (*p*_BH_ = 0.218 and *p*_BH_ = 0.112, respectively).

However, the relative percentages of neutrophils and lymphocyte did not differ between the groups (*p*_BH_ = 0.892 and *p*_BH_ = 0.915, respectively). Absolute and relative monocyte values were comparable between CMV groups, although monocyte percentages showed a non-significant nominal trend toward lower values in CMV-seropositive patients (*p*_adj_ = 0.086, *p*_BH_ = 0.313). Inflammatory ratios derived from leukocyte subsets, including NLR (*p*_BH_ = 0.900) and LMR (*p*_BH_ = 0.359), were similar between groups.

In contrast, the platelet-to-lymphocyte ratio (PLR) initially demonstrated a significantly lower value in CMV-seropositive patients at the univariable screening level (median 108.33 vs. 142.29; unadjusted *p* = 0.041). However, this association did not maintain statistical significance after controlling for confounders (*p*_adj_ = 0.388) and was further attenuated following multiple comparisons correction (*p*_BH_ = 0.704).

The erythrocyte sedimentation rate did not differ between groups (median 9 vs. 11 mm/h, *p*_BH_ = 0.788). All analyses were performed using the available case data.

### 4.3. Sociodemographic, Clinical, and Cognitive Characteristics According to CMV Serostatus

Sociodemographic, clinical, and cognitive characteristics according to CMV serostatus are presented in Table 5. Sociodemographic and illness-related variables were comparable between CMV-negative and CMV-positive patients. No significant differences were observed for age, age at illness onset, duration of the illness, or number of hospitalizations during the previous 12 months (all *p*_BH_ > 0.05). The psychopathological severity was also largely similar between the groups. PANSS total scores did not differ significantly between CMV-negative and CMV-positive patients (125.5 [102.75–145.25] vs. 137 [117–150], *p*_BH_ = 0.719).

Within the PANSS five-factor consensus model, the scores for the positive, negative, excited, and depressed factors were comparable between the groups (all *p*_BH_ > 0.05). In contrast, the disorganized/concrete factor initially demonstrated a significantly higher score in CMV-positive patients (15 [12–17]) compared to CMV-negative individuals (12.5 [9–16.25]; unadjusted *p* = 0.013). This association remained statistically significant even after adjusting for confounders (*p*adj = 0.031), although it did not survive the subsequent strict multiple comparison correction, shifting to a non-significant nominal trend following the Benjamini–Hochberg adjustment (*p*_BH_ = 0.181).

Cognitive measures were largely similar between groups. SCoRS Global Rating (8 [7–8.63] vs. 8.5 [8,9], *p*_BH_ = 0.705) and SCoRS Total Score (64 [55.75–69] vs. 70 [64–74], *p*_BH_ = 0.454) did not differ significantly between CMV-negative and CMV-positive patients. Domain-level analyses also showed no significant differences in memory, attention, executive functioning, language, or visuospatial ability (all *p*_BH_ > 0.05).

The lifetime number of suicide attempts initially differed between the groups, with higher values observed in CMV-negative patients (1 [0–1]) compared to CMV-positive individuals (0 [0–1]; unadjusted *p* = 0.034). Crucially, this association remained statistically significant after adjusting for confounders (*p*_adj_ = 0.030), demonstrating an independent nominal relationship; although, it did not survive the strict correction for multiple comparisons (*p*_BH_ = 0.219).

Diagnostic category (schizophrenia vs. schizophreniform disorder) was also compared between CMV-seropositive and CMV-seronegative patients and did not differ significantly (χ^2^ test, *p*_BH_ = 0.707).

### 4.4. Inflammatory Markers According to Sharp-Wave EEG Activity

Inflammatory markers according to the presence of sharp-wave EEG activity are presented in Table 6. Total leukocyte counts did not differ between patients with and without sharp waves (7 [5.82–8.49] vs. 6.98 [5.88–8.64] × 10^3^/µL, *p*_BH_ = 0.928). Similarly, no significant differences were observed for neutrophil counts or neutrophil percentages, nor for lymphocyte counts or lymphocyte percentages (all *p*_BH_ > 0.05).

In contrast, patients with sharp waves initially showed a significant elevation in monocyte profiles at the univariable level, demonstrating higher absolute monocyte counts (0.65 [0.55–0.76] vs. 0.56 [0.47–0.68] × 10^3^/µL; unadjusted *p* = 0.025) and higher monocyte percentages (9.2% [7.78–9.8%] vs. 8% [6.6–9.5%]; unadjusted *p* = 0.028). However, these associations did not maintain statistical significance after accounting for potential confounders, shifting to non-significant trends upon confounder adjustment (*p*_adj_ = 0.135, *p*_adj_ = 0.105) and multiple comparisons correction (*p*_BH_ = 0.302, *p*_BH_ = 0.253) for absolute counts and percentages, respectively.

The derived inflammatory ratios were comparable between the groups, including the neutrophil-to-lymphocyte ratio (NLR), the lymphocyte-to-monocyte ratio (LMR), and the platelet-to-lymphocyte ratio (PLR) (all *p*_BH_ > 0.05).

Finally, the erythrocyte sedimentation rate initially demonstrated a significantly higher value in patients with sharp waves (12.5 [10–14.75] vs. 7 [4–12.75] mm/h; unadjusted *p* = 0.010). However, this association was completely attenuated after accounting for potential confounders (*p*_adj_ = 0.607) and further eliminated following multiple comparisons correction (*p*_BH_ = 0.766).

### 4.5. Clinical and Cognitive Characteristics According to the Presence of Spontaneous Sharp-Wave EEG Activity

The clinical and cognitive characteristics according to the presence of spontaneous sharp-wave EEG activity are presented in Table 7. Patients with and without sharp-wave activity did not differ significantly in age, age at illness onset, duration of the illness, or number of psychiatric hospitalizations during the previous 12 months (all *p*_BH_ > 0.05).

Regarding psychopathological severity, patients exhibiting sharp-wave activity initially showed significantly lower PANSS total scores compared with those without sharp waves (125.50 [102.75–145.25] vs. 137.00 [117.00–150.00]; unadjusted *p* = 0.022). This difference remained statistically significant after adjusting for potential confounders (*p*_adj_ = 0.014) but was attenuated to a non-significant nominal trend following strict multiple comparisons correction (*p*_BH_ = 0.067).

Within the PANSS five-factor consensus model, patients with sharp waves initially demonstrated significantly lower scores on the disorganized/concrete factor (12.50 [9.00–16.25] vs. 15.00 [12.00–17.00]; unadjusted *p* = 0.047) and the excited factor (14.00 [10.00–22.25] vs. 20.00 [15.00–24.00]; unadjusted *p* = 0.019). Notably, both associations remained robust and further strengthened after controlling for potential confounders, yielding significant results for the disorganized/concrete factor (*p*_adj_ = 0.041) and the excited factor (*p*_adj_ = 0.017). However, these findings did not survive the strict multiplicity adjustment, shifting to non-significant nominal trends following Benjamini–Hochberg correction (*p*_BH_ = 0.131 and *p*_BH_ = 0.071, respectively).

No significant differences were observed for the positive, negative, or depressed factors (all *p*_BH_ > 0.05).

Cognitive functioning assessed using the Schizophrenia Cognition Rating Scale (SCoRS) also differed between groups. Patients with sharp-wave activity initially showed significantly lower SCoRS global rating scores (8.00 [7.00–8.63] vs. 8.50 [8.00–9.00]; unadjusted *p* = 0.002) and lower SCoRS total scores (64.00 [55.75–69.00] vs. 70.00 [64.00–74.00]; unadjusted *p* < 0.001). Importantly, both cognitive impairments remained statistically significant after adjusting for potential confounders, confirming independent associations for the global rating (*p*_adj_ = 0.012) and total scores (*p*_adj_ = 0.004). Following strict multiple comparisons correction, these findings were attenuated to strong, near-significant nominal trends (*p*_BH_ = 0.067 and *p*_BH_ = 0.060, respectively).

Exploratory domain-level analyses suggested initially lower impairment scores in patients with sharp-wave activity across all examined cognitive functions: memory (2.80 [2.15–3.20] vs. 3.20 [2.80–3.60]; unadjusted *p* < 0.001), attention (3.75 [3.44–3.75] vs. 4.00 [3.75–4.00]; unadjusted *p* < 0.001), executive functioning (3.60 [3.20–3.85] vs. 3.80 [3.60–4.00]; unadjusted *p* = 0.008), and language (3.13 [2.69–3.31] vs. 3.50 [3.00–3.75]; unadjusted *p* = 0.003). Additionally, these four domain-level associations remained stable after adjusting for potential confounders confirming independent differences for memory (*p*_adj_ = 0.001), attention (*p*_adj_ = 0.010), language (*p*_adj_ = 0.010), and executive functioning (*p*_adj_ = 0.026). Following multiple comparisons correction, the memory domain successfully maintained its statistical significance (*p*_BH_ = 0.028), whereas attention (*p*_BH_ = 0.075), language (*p*_BH_ = 0.093), and executive functioning (*p*_BH_ = 0.095) were attenuated to solid nominal trends.

Meanwhile, visuospatial ability did not differ significantly between groups (2.50 [1.88–3.00] vs. 2.50 [2.00–3.00], *p*_BH_ = 0.333).

The distribution of diagnostic categories did not differ significantly between patients with and without spontaneous sharp-wave activity (Fisher’s exact test, *p*_BH_ = 1.000), with a negligible effect size (φ = 0.01). Among patients presenting sharp waves, 28 (87.50%) were diagnosed with schizophrenia and four (12.50%) with schizophreniform disorder. Similarly, in the group without sharp waves, 78 (86.67%) had schizophrenia and 12 (13.33%) had schizophreniform disorder.

### 4.6. EEG Abnormalities According to Treatment-Resistant Status

The treatment-resistant status was examined in relation to electroencephalographic abnormalities (see Table 8), with effect sizes quantified using Cramér’s V coefficients. Associations were evaluated using chi-square tests, with Yates continuity correction applied when appropriate, and the resulting *p*-values were subsequently adjusted using the Benjamini–Hochberg procedure to control for the false discovery rate.

No significant differences were observed between treatment-resistant and non-treatment-resistant patients across any of the background rhythm characteristics, including dominant EEG rhythm, alpha peak frequency, spatial distribution of the dominant rhythm, bilateral symmetry, spindle-like activity of the dominant rhythm, and alpha activity with sharply contoured morphology (all *p*_BH_ = 0.999).

Similarly, beta activity parameters did not differ significantly between groups, including the presence, amplitude distribution, distribution pattern, and spindle-like activity of beta waves (all *p*_BH_ = 0.999), as well as beta localization (*p*_BH_ = 0.892).

Within irritative and epileptiform EEG abnormalities, spontaneous sharp-wave activity recorded during resting-state EEG was initially significantly more frequent among treatment-resistant patients (14 [35.90%]) compared with non-treatment-resistant individuals (12 [14.46%]; unadjusted *p* = 0.014). However, this pronounced univariable difference did not maintain statistical significance following multiple comparisons adjustment (*p*_BH_ = 0.406).

Likewise, sharp-wave activity overall was initially significantly associated with treatment resistance, being present in 16 (41.00%) treatment-resistant patients and 17 (20.50%) non-treatment-resistant patients (unadjusted *p* = 0.030). However, this baseline association did not remain statistically significant after applying the Benjamini–Hochberg correction (*p*_BH_ = 0.435).

In contrast, irritative EEG abnormalities showed a non-significant trend (6 [15.38%] vs. 4 [4.82%]; *p*_BH_ = 0.996), and no significant associations were observed for the remaining irritative, epileptiform, or slow-wave activity parameters (all *p*_BH_ > 0.05).

Overall, while baseline univariable analyses suggested that the prevalence of sharp-wave activity—particularly spontaneous sharp-wave discharges—differed between groups, this directional trend did not withstand strict multiplicity correction. Nevertheless, background rhythm organization, beta activity characteristics, and other epileptiform or slow-wave abnormalities showed no relationship with treatment-resistant status in either univariable or corrected models.

## 5. Discussion

The present study investigated the co-occurrence of cytomegalovirus serological status, electroencephalographic characteristics, inflammatory markers, and clinical–cognitive features in patients with psychotic disorders within a neuroimmune framework. Participants were recruited consecutively during hospital admission, reflecting routine clinical practice. CMV-seropositive and CMV-seronegative patients were comparable with respect to demographic characteristics, duration of the illness, diagnostic distribution, and overall psychopathological severity, supporting balanced group composition and reducing the likelihood of major confounding related to illness chronicity or clinical status. Within this multidimensional framework, the analyses examined parallel profiles related to CMV across electrophysiological, immunological, and clinical–cognitive domains. In addition, exploration analyses focused on the presence of sharp-wave activity as a specific electrophysiological feature that overlaps with immune signaling and clinical–cognitive variability.

Crucially, given the cross-sectional design of this study, these data capture a single point in time and cannot establish temporal precedence or causal directions. The observed patterns are discussed as non-causal associations and synchronized manifestations, underscoring the necessity of prospective, longitudinal studies to untangle potential causal pathways or shared third-variable influences.

### 5.1. Neurophysiological Findings in Relation to the Serostatus of CMV

Most EEG parameters reflecting background rhythm organization, beta activity, and slow-wave activity did not differ according to CMV serostatus, suggesting comparable electrophysiological patterns across groups. Dominant rhythm distribution, alpha peak frequency, spatial organization of the dominant rhythm, and bilateral symmetry remained within physiological limits in both groups, consistent with intact corticothalamic network function and stable oscillatory synchronization described in established neurophysiological models [7,8,44,45]. These observations align with contemporary conceptualizations of schizophrenia as a disorder of functional dysconnectivity rather than diffuse cortical disorganization [5,46,47], and are consistent with classical EEG studies reporting largely preserved background activity accompanied by localized or variable abnormalities rather than generalized disruption [48].

Beta activity was predominantly frontocentral and highly prevalent across recordings, in agreement with previous EEG investigations in schizophrenia. In the literature, this specific electrophysiological pattern is robustly linked to the effects of ongoing psychotropic medications—particularly antipsychotics and antiepileptic drugs—or non-specific cortical arousal states [9,10,11]. Given our naturalistic study design, the lack of statistical divergence after strict multiplicity control (*p*_BH_ > 0.05) indicates a homogenous background oscillatory footprint between the CMV subgroups. While qualitative visual analysis limits the detection of subtle power spectrum differences, these descriptive findings demonstrate that baseline cortical excitation did not differ across serostatus categories. This lack of variance in background activity suggests that the cohort maintained a uniform pharmacological and clinical baseline across viral subgroups, providing a stable foundation for the exploratory evaluation of other paroxysmal features.

In our univariable analysis, nominal differences within paroxysmal graphoelements were restricted to sharply contoured alpha activity and spontaneous resting-state sharp-wave discharges, which were both descriptively more frequent in the CMV-seronegative subgroup. Neither pattern, however, retained statistical significance following the Benjamini–Hochberg adjustment (*p*_BH_ = 0.435 and *p*_BH_ = 0.087, respectively). Although this lack of robust significance after multiple-testing correction requires a very conservative interpretation, the marginal trend observed for spontaneous sharp waves (*p*_BH_ < 0.10) provides a basis for exploratory neurophysiological discussion. Rather than indicating focal epileptic pathology, isolated sharp waves during wakeful rest in schizophrenia are understood as transient expressions of local excitatory–inhibitory (E/I) microcircuit imbalance [6,12]. In CMV-seronegative patients, the baseline neurodevelopmental deficit in inhibitory gating—frequently linked to NMDA receptor hypofunction on GABAergic interneurons—likely expresses itself directly as synchronized, unbuffered glutamatergic paroxysmal activity [5,6]. In contrast, latent cytomegalovirus infection in the seropositive group interacts with the host immune continuum to maintain persistent, low-grade neuroinflammation [22]. At the molecular level, this chronic viral latency stimulates resident microglia and brain endothelial cells to release pro-inflammatory cytokines, particularly interleukin-6 (IL-6) and tumor necrosis factor-alpha (TNF-α) [3,15,19]. Chronic exposure to elevated TNF-α and IL-6 signaling is known to induce homeostatic synaptic scaling, where glial-derived TNF-α drives the internalization of postsynaptic AMPA receptors and prolonged IL-6 downregulates phasic glutamatergic currents [15,17,18]. This ongoing cytokine signaling may effectively downscale or stabilize cortical microcircuit excitability. By suppressing the network’s capacity to generate rapid, highly synchronized paroxysmal discharges [5,44], this immune-mediated mechanism might mask the underlying electrophysiological deficits on the resting EEG, even in the presence of more severe clinical disorganization. Due to the cross-sectional nature of our study, these co-occurring profiles remain non-causal observations that require longitudinal tracking to map the precise cytokine-neurophysiological pathways.

The descriptive predominance of these resting-state sharp-wave discharges, rather than hyperventilation-induced paroxysms, offers an interesting point for discussion. While hyperventilation primarily triggers physiological metabolic and cerebrovascular modulations of cortical excitability, resting-state recordings are thought to reflect intrinsically generated cortical dynamics emerging from thalamo-cortical loops and large-scale oscillatory synchronization [7,44,45]. The nominal clustering of these graphoelements during rest may therefore point toward endogenous fluctuations within intrinsic functional networks, rather than externally driven or non-specific physiological reactivity. Understood this way, such isolated sharp waves could be viewed as transient departures from stable oscillatory regimes [14]. Although these patterns did not survive multiple-testing correction, this statistical trend is conceptually compatible with established dysconnectivity models of schizophrenia, where a failure in synaptic gating permits brief, localized hypersynchronous events without necessarily disrupting global network efficiency [5].

Descriptive examination of the spatial distribution of sharp-wave activity suggested predominantly bilaterally synchronous centrotemporal patterns, whereas generalized or strictly focal configurations were uncommon. Although these localization observations were exploratory and were not subjected to formal inferential tests, the overall spatial distribution may be more compatible with the involvement of associative cortical systems than with diffuse cortical dysfunction or primary epileptiform pathology. In this context, the predominance of centrotemporal patterns may tentatively suggest participation of temporo-parietal associative networks implicated in large-scale integrative processing [46,47]. In line with this network-level perspective, convergent evidence from recent magnetoencephalography studies has shown altered sharp-wave ripple organization and reduced large-scale network integration in schizophrenia [49]. This provides a useful conceptual parallel, suggesting that our descriptive observations may reflect localized disruptions in network coordination that align with known clinical variations in symptom dimensions.

From a broader perspective, while prior investigations of CMV exposure in schizophrenia have largely focused on structural imaging, cognition, or immunological measures, electrophysiological correlates have remained comparatively understudied. However, associations between CMV exposure and cognitive variability have been reported in both general and psychiatric populations, suggesting potential influences on large-scale brain networks involved in cognitive processing [29,50,51]. When considered alongside dysconnectivity models of schizophrenia that emphasize altered neural synchrony and large-scale integration [5,46,47], the present EEG findings are conceptually compatible with the hypothesis that exposure to CMV might subtly modulate transient network dynamics rather than disrupting global cortical organization. This points toward a potential convergence between immune-related processes and electrophysiological variability throughout the schizophrenia spectrum disorders [52].

### 5.2. CMV-Associated Immune Remodeling Patterns in the Context of Low-Grade Inflammation

A potentially counterintuitive finding in the present study was the higher prevalence of sharp-wave activity among CMV-seronegative patients, occurring alongside hematological patterns in CMV-seropositive individuals that are broadly consistent with immune adaptations described in latent CMV infection [53]. CMV seropositivity was observed in 87.29% of participants, closely matching global adult seroprevalence estimates of approximately 83% [54]. Previous studies indicate that CMV prevalence in schizophrenia-spectrum populations does not substantially differ from that of the general population [52], supporting the epidemiological plausibility of the present sample and suggesting that CMV exposure represents a common biological background rather than illness-specific enrichment.

After adjusting for clinical confounders—including age, sex, smoking status, and treatment resistance—and applying the Benjamini–Hochberg (BH) correction, CMV-seropositive patients demonstrated significantly higher total leukocyte counts compared to the seronegative group (*p*_BH_ = 0.045). Interestingly, this global leukocyte elevation was independently mirrored by absolute neutrophil and lymphocyte counts at the confounder-adjusted level (*p*_adj_ = 0.023 and *p*_adj_ = 0.008, respectively); however, these specific subsets did not survive the conservative false discovery rate control, shifting to non-significant trends after multiple testing correction (*p*_BH_ = 0.218 and *p*_BH_ = 0.112).

This robust, corrected elevation in total leukocytes remains highly consistent with the peripheral immune alterations and long-term shifts in immune cell architecture typically described in latent CMV infection [23,24,55].

Persistent antigenic stimulation during viral latency is well-known to drive the expansion of differentiated memory compartments without necessarily triggering overt, acute inflammatory activation [23,53,55].

This interpretation is strongly supported by the stability of our other hematological measures; both the relative percentages of these subsets and the derived inflammatory ratios, such as the neutrophil-to-lymphocyte ratio (*p*_BH_ = 0.900), remained remarkably similar between groups. Because these ratios are established markers of systemic immune balance rather than acute signaling [56,57], their equivalence—alongside unchanged erythrocyte sedimentation rate values [58]—reinforces the idea that CMV seropositivity in these patients reflects a stable, chronic homeostatic adaptation to viral latency rather than active, acute systemic inflammation.

Furthermore, while the platelet-to-lymphocyte ratio (PLR) initially appeared lower among CMV-seropositive patients at the univariable screening level, this trend was entirely attenuated after controlling for confounders (*p*_adj_ = 0.388) and multiple comparisons correction (*p*_BH_ = 0.704). This lack of adjusted significance, alongside the unchanged erythrocyte sedimentation rate values (*p*_BH_ = 0.788), further counters the hypothesis of active or overt inflammatory activation in our cohort. Instead, these observations align with the broader concept that platelet-related indices in chronic conditions reflect a complex interplay between systemic homeostatic factors rather than specific virus-induced pathology [56,59]. Collectively, the absence of robust differences in these peripheral inflammatory markers remains highly compatible with a maintained state of immune homeostasis, which is characteristic of a well-regulated immune adaptation during latent CMV infection [59,60].

Within schizophrenia research, these specific hematological patterns—characterized by a robust elevation in total leukocytes alongside stable inflammatory ratios and sedimentation rates—are broadly consistent with the low-grade inflammation hypothesis, which proposes a state of chronic, regulated immune activation rather than acute inflammatory signaling [19,61]. Persistent infectious exposures, such as latent CMV, can contribute to this long-term modulation of peripheral immune architecture, which may in turn influence neuroimmune signaling pathways [29,31]. Although peripheral markers provide only indirect indices of central processes, extensive evidence supports bidirectional immune–brain communication through cytokine-mediated, neural, and neurovascular mechanisms [15]. This framework offers a plausible biological pathway through which chronic immune remodeling might coincide with electrophysiological variability. Within this perspective, prior viral exposure may act as a modulatory biological context influencing neuroimmune signaling rather than as a direct pathogenic driver, potentially shaping subtle variations in network excitability and oscillatory coordination within already vulnerable neural systems.

### 5.3. Clinical and Cognitive Correlations of CMV Exposure

The clinical severity and global cognitive performance were largely comparable between the CMV groups, indicating that exposure to CMV is unlikely to determine the overall clinical phenotype of psychotic disorders [29]. After adjusting for key clinical confounders—including age, sex, smoking status, and treatment resistance—and applying strict Benjamini–Hochberg (BH) corrections, no significant differences were observed in global psychopathological severity (PANSS total score) or overall functional cognition (SCoRS global rating and total score).

Regarding specific dimensions, the initially higher scores on the disorganized/concrete factor of the PANSS among CMV-seropositive patients (*p*_adj_ = 0.031) shifted to a non-significant nominal trend following multiple-testing correction (*p*_BH_ = 0.181). Similarly, the domain-level item groupings (encompassing memory, attention, executive functioning, and language) revealed no discernible differences based on CMV status (all *p*_BH_ > 0.05). Consistent with our methodological framework, these item-level aggregations were treated strictly as descriptive and exploratory indices rather than validated psychometric subscales, preventing any definitive conclusions regarding isolated cognitive dimensions.

Given that these clinical variations did not retain statistical significance after multiplicity adjustment, these findings point toward subtle dimensional variations rather than a stark clinical divergence. Within this perspective, the frequent sharp-wave activity observed in the CMV-seronegative group may reflect a state of neural hyper-reactivity or alternative compensatory synchronization, where vulnerable networks actively expend functional effort to maintain baseline clinical stability. Conversely, the lower occurrence of such paroxysmal EEG patterns in the CMV-seropositive cohort, alongside the lack of differences in the SCoRS global rating or total score, might tentatively reflect a subtle, chronic viral-induced exhaustion of these plastic compensatory mechanisms—a pattern potentially consistent with frontally mediated network mechanisms [62,63] that manifests nominally as a slight trend toward disorganization.

Previous studies examining CMV exposure in schizophrenia have reported heterogeneous findings, some identifying associations with persistent negative symptom profiles [28] and others describing selective cognitive effects without consistent relationships with overall symptom severity [29]. The absence of negative symptom differences in the present study further underscores the variability between schizophrenia cohorts and supports the interpretation of CMV exposure as a modulatory rather than a phenotype-defining factor.

An additional observation was the initially higher lifetime number of suicide attempts among CMV-seronegative patients. Although this association remained independent at the confounder-adjusted level (*p*_adj_ = 0.030), it did not survive the strict multiple comparisons restriction, shifting to a non-significant nominal trend following the Benjamini–Hochberg adjustment (*p*_BH_ = 0.219). Given that this pattern did not maintain robust statistical significance, it should be interpreted with high conservatism and does not support a definitive association between CMV serostatus and suicidal behavior within schizophrenia spectrum disorders. Instead, this nominal variation likely reflects the broader clinical heterogeneity inherent to chronic psychiatric cohorts or the influence of unmeasured background factors not directly related to viral exposure.

Taken together, these findings are compatible with models proposing that immune-related processes associated with CMV exposure may be related to variability in functional neural dynamics without evidence of generalized clinical deterioration [3,5,15]. In particular, the absence of differences between schizophrenia and schizophreniform disorder in CMV serostatus or sharp-wave prevalence further suggests that these associations may operate across the schizophrenia spectrum independently of illness duration.

### 5.4. Sharp-Wave Activity and Its Clinical, Immune, and Treatment-Related Correlates

To fully understand the interplay between chronic viral exposure and neurophysiological alterations, it was essential to investigate whether the observed sharp-wave activity was driven by a parallel state of active peripheral inflammation. Regarding the relationship between peripheral inflammation and neurophysiological patterns, our adjusted analyses reveal an absence of robust inflammatory signatures associated with abnormal EEG activity, further reinforcing the non-destructive, modulatory nature of these paroxysmal patterns. While sharp-wave activity was initially associated with increased absolute monocyte counts (*p* = 0.025) and monocyte percentages (*p* = 0.028), as well as an elevated erythrocyte sedimentation rate (*p* = 0.010) at the univariable screening level, these findings did not maintain significance after controlling for covariates. Specifically, the differences in monocyte profiles were heavily attenuated upon confounder adjustment (*p*_adj_ = 0.135 for absolute counts; *p*_adj_ = 0.105 for percentages) and eliminated after multiple comparisons correction (*p*_BH_ = 0.302 and *p*_BH_ = 0.253, respectively). Similarly, the initial divergence in ESR dissolved after accounting for confounders (*p*_adj_ = 0.607) and Benjamini–Hochberg correction (*p*_BH_ = 0.766). This loss of statistical significance precisely highlights that the univariable variations were driven by secondary confounding factors—such as psychotropic medication regimens—rather than an active systemic inflammatory process. Instead, in this context, convergent neuroimmunological evidence indicates that peripheral immune signaling may influence brain function through multiple, more subtle immune–brain communication pathways [15], including modulation of microglial activity [17,18], with downstream effects on synaptic regulation and excitation–inhibition balance [64]. Peripheral immune activation has previously been associated with transient functional alterations in brain activity [15,65], which, given the sensitivity of thalamo-cortical oscillatory systems to neuromodulatory influences [7], may manifest itself as reversible EEG changes without evidence of diffuse cortical disruption.

On the other hand, to fully clarify the clinical relevance of these electrophysiological patterns and determine whether they reflect structural deficit or functional adaptation, it was essential to map sharp-wave activity directly against psychopathological and cognitive dimensions. In parallel with the observed neuroimmune trends, patients exhibiting sharp-wave activity demonstrated significantly lower psychopathological severity and superior cognitive functioning, suggesting that these electrophysiological features align with clinical and cognitive preservation rather than functional decline. Although the lower global PANSS scores (*p*_adj_= 0.014; *p*_BH_ = 0.067), as well as the reduced disorganized (*p*_adj_ = 0.041) and excited (*p*_adj_ = 0.017) factors, were attenuated to solid nominal trends after multiplicity correction, the independent associations with better cognitive performance remained remarkably resilient. Specifically, lower functional cognitive impairment was highly robust for both the SCoRS global rating (*p*_adj_ = 0.012; *p*_BH_ = 0.067) and total scores (*p*_adj_ = 0.004; *p*_BH_ = 0.060). Most notably, within the exploratory domain-level analyses, the memory domain retained its statistical significance even after strict Benjamini–Hochberg correction (*p*_adj_ = 0.001; *p*_BH_ = 0.028), while attention, language, and executive functioning persisted as strong, near-significant nominal trends.

This coherent clustering of lower symptom severity preserved memory, and the absence of systemic inflammation points toward our working hypothesis: rather than indicating passive brain dysfunction, spontaneous sharp waves might represent an active, resource-demanding framework of neural hyper-reactivity or alternative compensatory synchronization. Specifically, when primary thalamo-cortical networks face vulnerability, the brain may hypothetically recruit alternative, highly synchronized neural clusters to actively maintain the efficiency of information processing. This heightened, energy-consuming electrical activity could produce paroxysmal discharges as a functional byproduct, potentially serving as an active buffering mechanism that shields critical domains—such as memory—from clinical deterioration. Crucially, the higher prevalence of these compensatory sharp waves in CMV-seronegative patients suggests that the absence of chronic, virus-induced network wear might leave the necessary neuroplastic resources intact to mount such an energy-demanding functional defense. Conversely, in CMV-seropositive patients, this neuroplastic capacity could be constrained by a state of persistent immune activation, a mechanism indirectly aligned with our finding of significantly elevated total leukocyte counts in this group. This pattern is broadly consistent with theoretical models that propose that moderate neural variability may support adaptive processing and cognitive flexibility [14,66,67,68]. In addition, sleep EEG studies linking thalamo-cortical oscillations to cognitive and executive functions further support the interpretation of discrete electrophysiological signatures as potential indicators of circuit-level functional efficiency [69]. Consequently, these findings suggest that paroxysmal EEG activity may serve as a marker of preserved neuroplastic capacity, enabling vulnerable networks to actively expend functional effort to maintain baseline clinical and cognitive stability.

Beyond these clinical and cognitive correlates, the potential relationship between electrophysiological abnormalities and treatment-resistant status was critically evaluated. At the univariable screening level, resting-state spontaneous sharp-wave activity (*p* = 0.014) and overall sharp waves (*p* = 0.030) appeared initially more frequent among treatment-resistant patients. However, within our cross-sectional framework, these directional differences did not withstand strict multiplicity adjustment, shifting to entirely non-significant values after Benjamini–Hochberg correction (*p*_BH_ = 0.406 and *p*_BH_ = 0.435, respectively), while global EEG background organization remained thoroughly preserved (*p*_BH_ = 0.999). This loss of robust significance after controlling for the false discovery rate is of particular interest;; it indicates that the univariable trends may reflect secondary covariance—such as the heavy burden of psychotropic polypharmacy or clozapine treatment—rather than a stable, intrinsic neurobiological marker of treatment resistance.

Consequently, the absence of a resilient, independent association with treatment resistance aligns with our previous finding that these paroxysmal features do not track with diffuse cortical dysfunction or overall symptom exacerbation. Instead, within the boundaries of a cross-sectional design, these patterns are compatible with models suggesting localized, transient network instability related to glutamatergic excitation–inhibition imbalance in schizophrenia [1,16], rather than an indicator of irreversible therapeutic failure. Previous electrophysiological observations have similarly described such selective paroxysmal occurrences alongside a preserved background architecture [10,70]. While clozapine-related effects are well-known to induce electroencephalographic shifts, they may also overlap with the intrinsic modulation of disrupted neural synchrony characteristic of the illness [1,5,16]. Because sharp-wave activity was not independently tied to treatment-resistant status or systemic inflammation in our fully corrected models, these dynamics might be conceptualized as indicators of fluctuating micro-circuit adaptations. This provides a clearer, non-contradictory perspective on the underlying neurobiology, contributing to the broader discussion of schizophrenia as a condition involving highly heterogeneous neurobiological subtypes [2,71].

Taken together, the convergence of electrophysiological, immunological, and clinical findings in the present study suggests that CMV serostatus may reflect a distinct biological context associated with specific variations in neural network dynamics and systemic immune activation within the schizophrenia spectrum, rather than serving as a direct determinant of overt, generalized neuropathology. Within this cross-sectional framework, the presence or absence of this viral marker appears to align with different pathways of network adaptation, potentially reflecting differences in the availability of the neuroplastic resources required to maintain baseline clinical and cognitive stability.

## 6. Strengths and Limitations

This study integrates electrophysiological, immunological, clinical and cognitive data within a single well-characterized hospital-based cross-sectional cohort of patients with schizophrenia spectrum disorders. To our knowledge, it represents one of the first systematic investigations examining the serostatus of cytomegalovirus in relation to detailed qualitative EEG graphoelements in psychotic disorders. By combining these complementary domains, the study provides a broader perspective on possible links between CMV serostatus, immune markers, electrophysiological patterns, and clinical characteristics. Integration of electrophysiological and immune measures also allows observed associations to be conceptualized within a coherent neuroimmune framework. In addition, consecutive recruitment under routine clinical conditions enhances ecological validity by reflecting the variability typically encountered in real-world psychiatric practice.

Several limitations should also be considered when interpreting these findings. The cross-sectional design does not allow causal inference and prevents determining temporal relationships between CMV exposure, immune alterations, and electrophysiological characteristics. CMV exposure was assessed using IgG serological status, which reflects prior infection and is consistent with latent viral infection, but does not provide information on viral reactivation or current viral activity. Furthermore, peripheral inflammatory markers represent indirect indicators of neuroimmune processes and may not fully reflect immune activity within the central nervous system.

Another limitation is that our neurophysiological data came from routine visual electroencephalography rather than automated spectral analysis or digital quantitative EEG (qEEG) workflows. Although manual chart reviewing by a single blinded expert evaluator ensures methodological consistency across the entire cohort, this qualitative strategy still introduces an unavoidable element of subjectivity. Judgment ambiguities are well-documented phenomena in clinical neurophysiology, especially when assessing borderline or morphologically complex paroxysmal features like sharp waves. Incorporating computerized power spectrum metrics would have provided an objective, digitized benchmark to minimize individual grading biases, while potentially uncovering discrete changes in oscillatory power that escape visual inspection alone. While our data accurately captures real-world diagnostic patterns under uniform evaluation, future longitudinal studies using standardized, quantitative electrophysiological frameworks are necessary to confirm these exploratory observations.

Another constraint is the unequal distribution of our sample, with a relatively small CMV-seronegative subgroup. It is worth noting, however, that this imbalance reflects the characteristically high CMV-seroprevalence rates typically documented in both the general population and schizophrenia spectrum cohorts. While this natural asymmetry is representative of real-world epidemiological patterns, the modest size of the seronegative arm inherently limits the statistical power available for detecting subtle clinical or electrophysiological variations between the groups. Consequently, while our findings provide valuable observations within a uniform clinical sample, they should be considered preliminary, and validation in larger cohorts remains a useful step to confirm these trends.

Additionally, potential selection bias related to the hospital-based nature of this prospective recruitment was mitigated by including both outpatients presenting for routine re-evaluation and acute inpatients under both voluntary and involuntary admission—with informed consent rigorously obtained from all participants or their legally authorized representatives—thereby capturing a wide spectrum of clinical severity. However, because participants were recruited from a single psychiatric center, the generalizability of the findings to different geographical or primary care settings remains to be confirmed. Regarding pharmacological confounders, the extensive prevalence of complex psychotropic polypharmacy within our sample precluded granular statistical control for every individual antipsychotic type or dosage. To account for this clinically, treatment-resistant status—defined strictly by the clinical requirement for clozapine therapy or electroconvulsive therapy (ECT)—was utilized as a robust proxy variable in our adjusted models. While this approach controls for the most significant treatment-related neurophysiological and immunological shifts, the potential residual confounding influence of diverse ongoing background medications cannot be entirely excluded.

Additionally, while our multivariate models successfully controlled for key confounders including age, sex, and smoking status, other relevant clinical variables such as disease duration and historical hospitalization burden were not formally included in the statistical adjustment. Although these factors did not differ significantly between our primary comparison groups, their potential residual influence on peripheral inflammatory profiles cannot be completely discounted and should be verified in future, larger-scale studies.

## 7. Conclusions and Future Directions

In conclusion, the present findings suggest that cytomegalovirus serostatus may be associated with subtle neurobiological variability within schizophrenia spectrum disorders through mechanisms potentially involving neuroimmune processes rather than overt neuropathology. By integrating CMV serostatus, peripheral immune markers, detailed EEG characterization, and dimensional clinical assessment within a single cross-sectional cohort, this study provides additional insight into the relationships among viral exposure, immune status, and cortical network dynamics.

CMV-seropositive patients exhibited higher leukocyte counts, consistent with previously described immune alterations associated with latent CMV infection. A descriptive difference in the frequency of resting-state sharp-wave activity was observed between CMV groups, with higher rates among CMV-seronegative individuals; however, this observation did not remain statistically significant after correction for multiple comparisons. Importantly, sharp-wave activity was not independently associated with peripheral inflammatory markers or treatment-resistant status. This lack of independent covariance suggests that the observed electrophysiological variability cannot be readily explained by peripheral immune measures alone, nor is it driven by secondary clinical factors such as refractory illness course or related psychotropic medication regimens.

Clinical and cognitive outcomes were largely comparable between CMV groups. CMV-seropositive patients showed descriptively higher scores on the disorganization symptom dimension, although this association did not remain statistically significant after correction for multiple comparisons. In separate analyses, patients exhibiting sharp-wave activity demonstrated better overall cognitive functioning and better performance within the memory-related item grouping. Given the limited psychometric validation of these domain-level groupings, these observations should be regarded as hypothesis-generating rather than evidence of specific cognitive effects.

Taken together, the present findings suggest that CMV exposure may represent a biological context associated with variability in immune and electrophysiological measures, while overall clinical severity, global cognitive functioning, and background EEG organization remained largely comparable between groups. The apparent dissociation between peripheral immune measures, sharp-wave activity, and clinical manifestations highlights the complexity of neuroimmune interactions in schizophrenia spectrum disorders and suggests that these relationships are unlikely to follow a simple linear pattern.

Because of the cross-sectional design, all observed associations should be interpreted as non-causal. Future longitudinal and multimodal studies incorporating repeated EEG assessments, quantitative electrophysiological analyses, cytokine profiling, and neuroimaging markers of neuroinflammation will be necessary to clarify the temporal and mechanistic relationships among CMV exposure, immune regulation, cortical network dynamics, and clinical outcomes.

## Figures and Tables

**Table 1:** Sociodemographic, clinical, cognitive and biological characteristics of the study sample (*N* = 123).

Characteristics	Variable		Median (IQR)/n (%)
A. Sociodemographic characteristics	Sex	Male	98 (79.67%)
		Female	25 (20.32%)
	Age (years)		42 (33–50)
	Educational level	Low educational level	20 (16.26%)
		Medium educational level	81 (65.85%)
		High educational level	22 (17.88%)
	Place of residence	Urban	99 (80.48%)
		Rural	24 (19.51%)
	Smoking status	Current smoker	79 (64.22%)
		Non-smoker	44 (35.77%)
B. Illness-related characteristics	Age at onset (years)		25 (20–34)
	Duration of illness (years)		11 (4–21)
	Hospitalizations in the last 12 months		2 (1–4)
	Lifetime number of suicide attempts		0 (0–1)
	Treatment-resistant illness		39 (31.71%)
	Diagnostic category (DSM-5-TR)	Schizophrenia	107 (86.99%)
		Schizophreniform disorder	16 (13.01%)
C. Psychopathological severity	PANSS total score		135 (114–149)
	Positive factor		22 (17–25)
	Negative factor		24 (18–29)
	Disorganized/Concrete factor		14 (10–17)
	Excited factor		19 (14–24)
	Depressed factor		12 (10–14)
D. Cognitive functioning (SCoRS)	SCoRS Global Rating		8.5 (7.5–9)
	SCoRS Total Score		69 (61–73)
	Memory		3.2 (2.6–3.4)
	Attention		4 (3.75–4)
	Executive Functioning		3.8 (3.4–4)
	Language		3.25 (3–3.5)
	Visuospatial Ability		2.5 (2–3)
E. Inflammatory and immune markers	Leucocytes (×1000/µL)		6.96 (5.86–8.63)
	Neutrophils (×1000/µL)		3.79 (3.08–4.96)
	Neutrophils (%)		55.9 (49.82–62.7)
	Lymphocytes (×1000/µL)		2.21 (1.78–2.77)
	Lymphocytes (%)		32.45 (25.68–38.13)
	Monocytes (×1000/µL)		0.59 (0.47–0.73)
	Monocytes (%)		8.3 (6.88–9.8)
	NLR		1.72 (1.29–2.50)
	LMR		3.84 (3.10–4.54)
	PLR		114.67 (80.78–146.08)
	ESR (mm/h)		10 (4–14)
F. Serological status of CMV	CMV-negative		15 (12.71%)
	CMV-positive		103 (87.29%)

Note: Values are presented as median (interquartile range [IQR]) for continuous variables and n (%) for categorical variables. CMV exposure was defined according to qualitative CMV IgG serological status. PANSS five-factor consensus model scores (positive, negative, disorganized/concrete, excited, and depressed factors) represent the sum of predefined PANSS items.

**Table 2:** Distribution of electroencephalographic characteristics in the study sample.

Characteristics	EEG Variable	Category	n (%)
A. Background rhythm characteristics	Dominant EEG rhythm	Alpha rhythm	118 (96.72)
		Slowed posterior rhythm (alpha–theta range)	3 (2.46)
		Theta rhythm	1 (0.82)
	Alpha peak frequency (Hz)	9.83 ± 1.1 (Mean ± SD)	
	Spatial distribution of dominant rhythm	Centrotemporal	57 (47.11)
		Parietotemporal	63 (52.07)
		Generalized	1 (0.83)
	Bilateral symmetry of dominant rhythm	Symmetric	107 (88.43)
		Asymmetric	14 (11.57)
	Spindle-like activity of the dominant rhythm	Present	49 (40.16)
	Alpha activity with sharply contoured morphology	Present	6 (4.92)
B. Beta activity characteristics	Presence of beta activity	Present	120 (98.36)
	Beta amplitude	Low amplitude	35 (29.17)
		Moderate amplitude	23 (19.17)
		High amplitude	62 (51.67)
	Beta localization	Frontocentral	100 (85.47)
		Generalized	14 (11.97)
		Other focal distributions	3 (2.56)
	Beta distribution pattern	Temporal	93 (94.89)
		Posterior	5 (5.10)
	Spindle-like beta activity	Present	1 (0.82)
C. Irritative and epileptiform EEG abnormalities	Irritative EEG abnormalities	Present	10 (8.20)
	Spontaneous sharp-wave activity (resting-state EEG)	Present	26 (21.31)
	Sharp waves during hyperventilation	Present	11 (9.01)
	Sharp-wave activity (overall)	Present	33 (27.04)
	Localization of sharp-wave activity	Centrotemporal	21 (63.64)
		Parietotemporal	6 (18.18)
		Centroparietotemporal	3 (9.09)
		Frontocentrotemporal	1 (3.03)
		Central	1 (3.03)
		Occipitotemporal (left)	1 (3.03)
	EEG features suggestive of a structural lesion	Present	13 (10.66)
	Epileptiform EEG abnormalities	Present	1 (0.82)
	Spontaneous spike discharges (resting-state EEG)	Present	3 (2.46)
	Spontaneous spike–wave complexes	Present	7 (5.74)
	Spike or spike–wave activity (overall)	Present	9 (7.38)
	Spike or spike–wave activity during hyperventilation	Present	3 (2.46)
	Spike or spike–wave activity during photic stimulation	Present	3 (2.46)
		Centrotemporal	4 (44.44)
		Parietotemporal	1 (11.11)
		Temporal	1 (11.11)
	Localization of spike and spike–wave activity	Occipital (right)	1 (11.11)
		Generalized	1 (11.11)
		Non-systematized	1 (11.11)
D. Slow-wave activity	Spontaneous rhythmic delta activity	Present	13 (10.66)
	Rhythmic delta activity (overall)	Present	17 (13.93)
	Hyperventilation-induced delta activity	Present	6 (4.92)
	Localization of delta activity	Frontocentral	4 (23.53)
		Centrotemporal	4 (23.53)
		Centroparietotemporal	2 (11.76)
		Temporal	2 (11.76)
		Occipital	2 (11.76)
		Generalized	2 (11.76)
		Non-systematized	1 (5.88)
	Hyperventilation-induced rhythmic theta activity	Present	6 (4.92)
	Spontaneous rhythmic theta activity	Present	20 (16.39)
	Rhythmic theta activity (overall)	Present	24 (19.67)
	Localization of theta activity	Centrotemporal	9 (40.91)
		Central	4 (18.18)
		Temporal	2 (9.09)
		Frontocentral	2 (9.09)
		Centroparietal	1 (4.55)
		Frontal	1 (4.55)
		Frontotemporal	1 (4.55)
		Parietotemporal	1 (4.55)
		Occipital	1 (4.55)
	Delta activity during eyes-closed recording and photic stimulation	Present	1 (0.82)

Note: Values are presented as the number of patients and percentage of the total EEG sample (*N* = 122). Continuous variables are presented as mean ± standard deviation (SD). EEG terminology and category definitions are consistent with those used in the main results analyses. Descriptive statistics include all participants who underwent EEG recording, irrespective of the availability of serological or laboratory data.

**Table 3:** Association between CMV IgG serostatus and EEG characteristics.

Characteristics	EEG Variable	Category	CMV− n (%)	CMV+ n (%)	*p*-Value (Cramér’s V)	BH Adj *p*-Value (*p*_BH_)
A. Background rhythm characteristics	Dominant EEG rhythm	Alpha rhythm	13 (86.67)	100 (98.04)	0.508 (0.061)	0.775
		Slowed posterior rhythm (alpha–theta range)	1 (6.67)	2 (1.96)		
		Theta rhythm	1 (6.67)	0 (0.0)		
	Alpha peak frequency (Hz)	8 Hz	4 (26.67)	8 (7.84)	0.128 (0.141)	0.464
		9 Hz	2 (13.33)	35 (34.31)		
		10 Hz	4 (26.67)	33 (32.35)		
		11 Hz	2 (13.33)	17 (16.67)		
		12 Hz	2 (13.33)	9 (8.82)		
	Spatial distribution of dominant rhythm	Centrotemporal	5 (33.33)	51 (50.00)	0.299 (0.096)	0.578
		Parietotemporal	9 (60.00)	50 (49.02)		
		Generalized	1 (6.67)	0 (0.0)		
	Bilateral symmetry of dominant rhythm	Symmetric	13 (86.67)	90 (88.24)	0.893 (0.012)	0.959
		Asymmetric	2 (13.33)	11 (10.78)		
	Spindle-like activity of the dominant rhythm	Present	4 (26.67)	43 (42.16)	0.253 (0.106)	0.667
	Alpha activity with sharply contoured morphology	Present	3 (20.00)	3 (2.94)	**0.030** † (0.201)	0.435
B. Beta activity characteristics	Presence of beta activity	Present	14 (93.33)	101 (99.02)	0.113 (0.147)	0.468
	Beta amplitude	Low amplitude	6 (40.00)	28 (27.45)	0.205 (0.117)	0.661
		Moderate amplitude	1 (6.67)	21 (20.59)		
		High amplitude	7 (46.7)	52 (50.98)		
	Beta localization	Frontocentral	10 (66.67)	85 (83.33)	0.376 (0.082)	0.682
		Generalized	3 (20.00)	11 (10.78)		
		Other focal distributions	1 (6.67)	2 (1.96)		
	Beta distribution pattern	Temporal	9 (60.00)	80 (78.43)	0.287 (0.098)	0.595
		Posterior	1 (6.67)	3 (2.94)		
	Spindle-like beta activity	Present	0 (0.0)	1 (0.98)	0.700 (0.036)	0.883
C. Irritative and epileptiform EEG abnormalities	Irritative EEG abnormalities		3 (20.00)	7 (6.86)	0.089 (0.157)	0.516
	Spontaneous sharp-wave activity (resting-state EEG)		8 (53.33)	17 (16.67)	**0.003** † (0.274)	0.087
	Sharp waves during hyperventilation		2 (13.33)	9 (8.82)	0.576 (0.052)	0.795
	Sharp-wave activity (overall)		8 (53.33)	24 (23.53)	**0.035** † (0.195)	0.338
	EEG features suggestive of a structural lesion		4 (26.67)	9 (8.82)	0.106 † (0.149)	0.512
	Epileptiform EEG abnormalities		0 (0.00)	1 (0.98)	0.700 (0.036)	0.846
	Spontaneous spike discharges (resting-state EEG)		1 (6.67)	2 (1.96)	0.282 (0.099)	0.629
	Spontaneous spike–wave complexes		1 (6.67)	6 (5.88)	0.905 (0.011)	0.937
	Spike or spike–wave activity (overall)		2 (13.33)	7 (6.86)	0.380 (0.081)	0.648
	Spike or spike–wave activity during hyperventilation		2 (13.33)	1 (0.98)	0.051† (0.180)	0.370
	Spike or spike–wave activity during photic stimulation		0 (0.00)	3 (2.94)	0.501 (0.062)	0.807
D. Slow-wave activity	Spontaneous rhythmic delta activity		3 (20.00)	10 (9.80)	0.241 (0.108)	0.699
	Rhythmic delta activity (overall)		3 (20.00)	14 (13.73)	0.520 (0.059)	0.754
	Hyperventilation-induced delta activity		1 (6.67)	5 (4.90)	0.772 (0.027)	0.896
	Hyperventilation-induced rhythmic theta activity		1 (6.67)	5 (4.90)	0.772 (0.027)	0.861
	Spontaneous rhythmic theta activity		2 (13.33)	18 (17.65)	0.679 (0.038)	0.895
	Rhythmic theta activity (overall)		3 (20.00)	21 (20.59)	0.958 (0.005)	0.958
	Delta activity during eyes-closed recording and photic stimulation		1 (6.67)	0 (0.00)	0.264 † (0.103)	0.638

Note: EEG variables were compared between CMV-seronegative (CMV−) and CMV-seropositive (CMV+) participants using chi-square tests. The values are presented as n (%), representing proportions within each CMV serological group. Yates continuity correction was applied when appropriate. Statistically significant associations (*p* < 0.05) are indicated in bold. † Yates continuity correction applied. Cramér’s V coefficients are reported as effect size measures. BH adj. *p*-value (*p*_BH_): *p*-value adjusted for multiple comparisons using the Benjamini–Hochberg procedure.

**Table 4:** Comparison of inflammatory and immune markers according to CMV serostatus.

Parameter	CMV-Negative	CMV-Positive	*p*-Value Adjusted for Confounders (*p*_adj_)	BH Adj *p*-Value (*p*_BH_)
Leucocytes (×1000/µL)	5.77 (5.38–6.49)	7.24 (6.33–9.29)	**0.002**	**0.045**
Neutrophils (×1000/µL)	3.35 (2.71–3.78)	4.01 (3.2–5.59)	**0.023**	0.218
Neutrophils (%)	57.4 (49.90–61.05)	55.8 (50–62.6)	0.646	0.892
Lymphocytes (×1000/µL)	1.75 (1.55–2.06)	2.3 (1.95–2.9)	**0.008**	0.112
Lymphocytes (%)	31 (26.95–36.5)	32.7 (25.6–38.2)	0.884	0.915
Monocytes (×1000/µL)	0.56 (0.44–0.62)	0.6 (0.5–0.75)	0.178	0.515
Monocytes (%)	9.6 (7.95–10.75)	8.2 (6.8–9.5)	0.086	0.313
NLR	1.78 (1.37–2.21)	1.72 (1.29–2.50)	0.621	0.900
LMR	3.49 (2.89–4.19)	3.91 (3.22–4.75)	0.111	0.359
PLR	142.29 (108.74–160.35)	108.33 (76.61–136.31)	0.388	0.704
ESR (mm/h)	11 (5.5–12)	9 (4–14)	0.652	0.788

Note: Values are presented as median (IQR). Comparisons were performed using the Mann–Whitney U test. Analyses used available case data. Significant results (*p* < 0.05) are shown in bold. BH adj. *p*-value (*p*_BH_): *p*-value adjusted for multiple comparisons using the Benjamini–Hochberg procedure. *p*-value adjusted for confounders (*p*_adj_): *p*-value adjusted for confounders (age, sex, smoking status, and treatment resistance) using general linear models.

**Table 5:** Comparison of sociodemographic, clinical, and cognitive characteristics according to CMV serostatus.

Parameter	CMV-Negative	CMV-Positive	*p*-Value	BH Adj *p*-Value (*p*_BH_)
Age (years)	45.5 (38.75–51)	41 (32–50)	0.344	0.713
Age at onset (years)	25 (20.75–36.25)	25 (19–34)	0.422	0.719
Duration of illness (years)	13.5 (6.75–21)	11 (3–22)	0.731	0.815
Hospitalizations in the last 12 months	2 (1–5)	2 (1–3)	0.605	0.923
PANSS total score	125.5 (102.75–145.25)	137 (117–150)	0.372	0.719
Positive factor	20.5 (12.25–24)	22 (18–25)	0.930	0.930
Negative factor	23 (19–26.25)	24 (18–30)	0.650	0.820
Disorganized/Concrete factor	12.5 (9–16.25)	15 (12–17)	**0.031**	0.181
Excited factor	14 (10–22.25)	20 (15–24)	0.335	0.746
Depressed factor	13 (11–15)	12 (10–14)	0.648	0.854
SCoRS Global Rating	8 (7–8.63)	8.5 (8–9)	0.437	0.705
SCoRS Total Score	64 (55.75–69)	70 (64–74)	0.188	0.454
Memory	2.8 (2.15–3.2)	3.2 (2.8–3.6)	0.083	0.400
Attention	3.75 (3.44–3.75)	4 (3.75–4)	0.767	0.824
Executive Functioning	3.6 (3.2–3.85)	3.8 (3.6–4)	0.679	0.788
Language	3.13 (2.69–3.31)	3.5 (3–3.75)	0.181	0.476
Visuospatial Ability	2.5 (1.88–3)	2.5 (2–3)	0.083	0.344
Lifetime number of suicide attempts	1 (0–1)	0 (0–1)	**0.030**	0.219

Note: Values are presented as median (IQR). Group differences were assessed using the Mann–Whitney U test. PANSS five-factor consensus model scores (positive, negative, disorganized/concrete, excited, and depressed factors) represent the sum of predefined PANSS items. Significant results (*p* < 0.05) are shown in bold. BH adj. *p*-value (*p*_BH_): *p*-value adjusted for multiple comparisons using the Benjamini–Hochberg procedure. *p*-value adjusted for confounders (*p*_adj_): *p*-value adjusted for confounders (age, sex, smoking status, and treatment resistance) using general linear models.

**Table 6:** Inflammatory markers according to the presence of sharp-wave EEG activity.

Parameter	Sharp Waves Present	Sharp Waves Absent	*p*-Value Adjusted for Confounders (*p*_adj_)	BH Adj *p*-Value (*p*_BH_)
Leucocytes (×1000/µL)	7 (5.82–8.49)	6.98 (5.88–8.64)	0.832	0.928
Neutrophils (×1000/µL)	3.9 (3.39–4.36)	3.78 (2.99–5.09)	0.869	0.900
Neutrophils (%)	58.65 (51.53–62.2)	54.95 (49.55–63.08)	0.596	0.785
Lymphocytes (×1000/µL)	2.22 (1.68–2.65)	2.23 (1.94–2.79)	0.865	0.930
Lymphocytes (%)	30.4 (24.88–36.08)	33.5 (26.15–38.3)	0.332	0.566
Monocytes (×1000/µL)	0.65 (0.55–0.76)	0.56 (0.47–0.68)	0.135	0.302
Monocytes (%)	9.2 (7.78–9.8)	8 (6.6–9.5)	0.105	0.253
NLR	1.88 (1.39–2.49)	1.64 (1.29–2.42)	0.807	0.936
LMR	3.61 (2.71–4.33)	3.93 (3.24–4.97)	0.074	0.216
PLR	125.64 (72.96–147.86)	110.14 (86.08–141.39)	0.432	0.626
ESR (mm/h)	12.5 (10–14.75)	7 (4–12.75)	0.607	0.766

Note: Values are presented as median (interquartile range, IQR). Between-group comparisons were performed using the two-tailed Mann–Whitney U test. The analyses were conducted using available case data. BH adj. *p*-value (*p*_BH_): *p*-value adjusted for multiple comparisons using the Benjamini–Hochberg procedure. *p*-value adjusted for confounders (*p*_adj_): *p*-value adjusted for confounders (age, sex, smoking status, and treatment resistance) using general linear models.

**Table 7:** Clinical and cognitive characteristics according to the presence of sharp-wave EEG activity.

Parameter	Sharp Waves Present	Sharp Waves Absent	*p*-Value Adjusted for Confounders (*p*_adj_)	BH Adj *p*-Value (*p*_BH_)
Age (years)	45.5 (38.75–51)	41 (32–50)	0.300	0.544
Age at onset (years)	25 (20.75–36.25)	25 (19–34)	0.341	0.549
Duration of illness (years)	13.5 (6.75–21)	11 (3–22)	0.762	0.920
Hospitalizations in the last 12 months	2 (1–5)	2 (1–3)	0.935	0.935
PANSS total score	125.5 (102.75–145.25)	137 (117–150)	**0.014**	0.067
Positive factor	20.5 (12.25–24)	22 (18–25)	0.077	0.203
Negative factor	23 (19–26.25)	24 (18–30)	0.190	0.368
Disorganized/Concrete factor	12.5 (9–16.25)	15 (12–17)	**0.041**	0.131
Excited factor	14 (10–22.25)	20 (15–24)	**0.017**	0.071
Depressed factor	13 (11–15)	12 (10–14)	0.415	0.634
SCoRS Global Rating	8 (7–8.63)	8.5 (8–9)	**0.012**	0.067
SCoRS Total Score	64 (55.75–69)	70 (64–74)	**0.004**	0.060
Memory	2.8 (2.15–3.2)	3.2 (2.8–3.6)	**0.001**	**0.028**
Attention	3.75 (3.44–3.75)	4 (3.75–4)	**0.010**	0.075
Executive Functioning	3.6 (3.2–3.85)	3.8 (3.6–4)	**0.026**	0.095
Language	3.13 (2.69–3.31)	3.5 (3–3.75)	**0.010**	0.093
Visuospatial Ability	2.5 (1.88–3)	2.5 (2–3)	0.161	0.333
Lifetime number of suicide attempts	1 (0–1)	0 (0–1)	0.453	0.625

Note: Values are presented as median (interquartile range, IQR). Continuous variables were compared using the two-tailed Mann–Whitney U test. PANSS five-factor consensus model scores (positive, negative, disorganized/concrete, excited, and depressed factors) represent the sum of predefined PANSS items. Statistically significant *p*-values (*p* < 0.05) are shown in bold. BH adj. *p*-value (*p*_BH_): *p*-value adjusted for multiple comparisons using the Benjamini–Hochberg procedure. *p*-value adjusted for confounders (*p*_adj_): *p*-value adjusted for confounders (age, sex, smoking status, and treatment resistance) using general linear models.

**Table 8:** Association between treatment-resistant illness and EEG abnormalities.

Characteristics	EEG Variable	Category	Treatment-Resistant n (%)	Non-Treatment-Resistant n (%)	*p*-Value (Cramér’s V)	BH Adj *p*-Value (*p*_BH_)
A. Background rhythm characteristics	Dominant EEG rhythm	Alpha rhythm	38 (97.44)	80 (96.39)	0.788 (0.025)	0.999
		Slowed posterior rhythm (alpha–theta range)	1 (2.56)	2 (2.41)		
		Theta rhythm	0 (0.0)	1 (1.20)		
	Alpha peak frequency	8 Hz	3 (7.69)	9 (10.84)	0.922 (0.009)	0.999
		9 Hz	14 (35.90)	24 (28.92)		
		10 Hz	12 (30.77)	28 (33.73)		
		11 Hz	7 (17.95)	13 (15.66)		
		12 Hz	3 (7.69)	8 (9.64)		
	Spatial distribution of dominant rhythm	Centrotemporal	17 (43.59)	40 (48.19)	0.726 (0.032)	0.999
		Parietotemporal	22 (56.41)	41 (49.40)		
		Generalized	0 (0.00)	1 (1.20)		
	Bilateral symmetry of dominant rhythm	Symmetric	35 (89.74)	72 (86.75)	0.752 (0.029)	0.999
		Asymmetric	4 (10.26)	10 (12.05)		
	Spindle-like activity of the dominant rhythm	Present	14 (35.90)	35 (42.17)	0.510 (0.061)	0.999
	Alpha activity with sharply contoured morphology	Present	2 (5.13)	4 (4.82)	0.941 (0.007)	0.999
B. Beta activity characteristics	Presence of beta activity	Present	39 (100.00)	81 (97.59)	0.328 (0.090)	0.999
	Beta amplitude	Low amplitude	10 (25.64)	25 (30.12)	0.682 (0.038)	0.999
		Moderate amplitude	7 (17.95)	16 (19.28)		
		High amplitude	22 (56.41)	40 (48.19)		
	Beta localization	Frontocentral	34 (87.18)	66 (79.52)	0.123 (0.143)	0.892
		Generalized	2 (5.13)	12 (14.46)		
		Other focal distributions	2 (5.13)	1 (1.20)		
	Beta distribution pattern	Temporal	30 (76.92)	63 (75.90)	0.888 (0.013)	0.999
		Posterior	2 (5.13)	3 (3.61)		
	Spindle-like beta activity	Present	0 (0.00)	1 (1.20)	0.491 (0.064)	0.999
C. Irritative and epileptiform EEG abnormalities	Irritative EEG abnormalities	Present	6 (15.38)	4 (4.82)	0.103 † (0.151)	0.996
	Spontaneous sharp-wave activity (resting-state EEG)	Present	14 (35.90)	12 (14.46)	**0.014** † (0.227)	0.406
	Sharp waves during hyperventilation	Present	3 (7.69)	8 (9.64)	0.726 (0.032)	0.999
	Sharp-wave activity (overall)	Present	16 (41.00)	17 (20.50)	**0.030** † (0.201)	0.435
	EEG features suggestive of a structural lesion	Present	5 (12.82)	8 (9.64)	0.595 (0.049)	0.999
	Epileptiform EEG abnormalities	Present	0 (0.00)	1 (1.20)	0.491 (0.064)	0.999
	Spontaneous spike discharges (resting-state EEG)	Present	1 (2.56)	2 (2.41)	0.959 (0.005)	0.993
	Spontaneous spike–wave complexes	Present	2 (5.13)	5 (6.02)	0.843 (0.018)	0.999
	Spike or spike–wave activity (overall)	Present	2 (5.13)	7 (8.43)	0.515 (0.060)	0.999
	Spike or spike–wave activity during hyperventilation	Present	1 (2.56)	2 (2.41)	0.959 (0.005)	0.959
	Spike or spike–wave activity during photic stimulation	Present	0 (0.00)	3 (3.61)	0.229 (0.111)	0.999
D. Slow-wave activity	Spontaneous rhythmic delta activity	Present	5 (12.82)	8 (9.64)	0.595 (0.049)	0.999
	Rhythmic delta activity (overall)	Present	7 (17.95)	10 (12.05)	0.380 (0.081)	0.999
	Hyperventilation-induced delta activity	Present	3 (7.69)	3 (3.61)	0.331 (0.090)	0.999
	Hyperventilation-induced rhythmic theta activity	Present	2 (5.13)	4 (4.82)	0.941 (0.007)	0.999
	Spontaneous rhythmic theta activity	Present	6 (15.38)	14 (16.87)	0.837 (0.019)	0.999
	Rhythmic theta activity (overall)	Present	7 (17.95)	17 (20.48)	0.743 (0.030)	0.999
	Delta activity during eyes-closed recording and photic stimulation	Present	0 (0.00)	1 (1.20)	0.491 (0.064)	0.999

Note: Values are presented as n (%). Between-group comparisons were performed using the chi-square test, with Yates continuity correction applied when appropriate. Statistically significant results (*p* < 0.05) are shown in bold. † Yates continuity correction applied. Cramér’s V coefficients are reported as effect size measures. BH adj. *p*-value (*p*_BH_): *p*-value adjusted for multiple comparisons using the Benjamini–Hochberg procedure.

## Data Availability

Due to the sensitive nature of the data and the absence of participant consent for public data sharing, the dataset generated and/or analyzed during the current study are not publicly available.
